# Collecting Duct Renal Cell Carcinoma Found to Involve the Collecting System During Partial Nephrectomy: A Case Report

**DOI:** 10.15586/jkcvhl.2015.37

**Published:** 2015-06-24

**Authors:** Andrew C Harbin, Brett A Styskel, Viren Patel, He Wang, Daniel D Eun

**Affiliations:** 1 Department of Urology; 2 Department of Pathology, Temple University Hospital, 3401 N, Broad Street, Philadelphia, PA 19147, USA.

## Abstract

Collecting duct carcinoma (CDC) is a rare and aggressive form of renal cell carcinoma (RCC) arising from the principal cells of the collecting duct. One third of cases present with metastatic disease, but many present in a manner similar to conventional RCC or urothelial carcinoma (UC). We discuss a case of CDC which presented as a small mass at the cortico-medullary junction, and was discovered at robotic partial nephrectomy (RPN) to be grossly involving the collecting system. A 62-year-old man presented with a small renal mass suspicious for RCC, which was found on computed tomography (CT) after an episode of gross hematuria. After thorough workup, RPN was attempted; however, intraoperatively the mass was found to be involving the collecting system. Radical nephroureterectomy was performed, and the pathology report revealed CDC. CDC is a rare and aggressive form of RCC. While many cases are metastatic at diagnosis, most patients present with the incidental finding of a small renal mass. There are no reports of a CDC involving the collecting system at RPN after negative ureteroscopy preoperatively. The adjuvant therapeutic options for CDC are limited, and long term survival is poor.

## Introduction

Collecting duct carcinoma (CDC), also known as Bellini duct carcinoma, is a rare form of renal cell carcinoma (RCC) that arises from cells of the collecting duct of the kidney. Although it represents less than one percent of all RCC cases (1), CDC is particularly aggressive, and up to 32% of cases may be metastatic at diagnosis (2). Typical presentation is similar to that of clear cell RCC (1, 3, 4), though symptoms from metastatic disease or paraneoplastic syndromes at presentation have been described (5–7).

We discuss a case of CDC that presented as a centrally located renal mass, not visible on ureterscopy but eventually found to be grossly invading the collecting system at the time of partial nephrectomy. Included are pathologic images and a review of the literature.

## Ethics approval

The following review of clinical data was performed after proper institutional review board approval.

## Case Report

**Figure 1.**
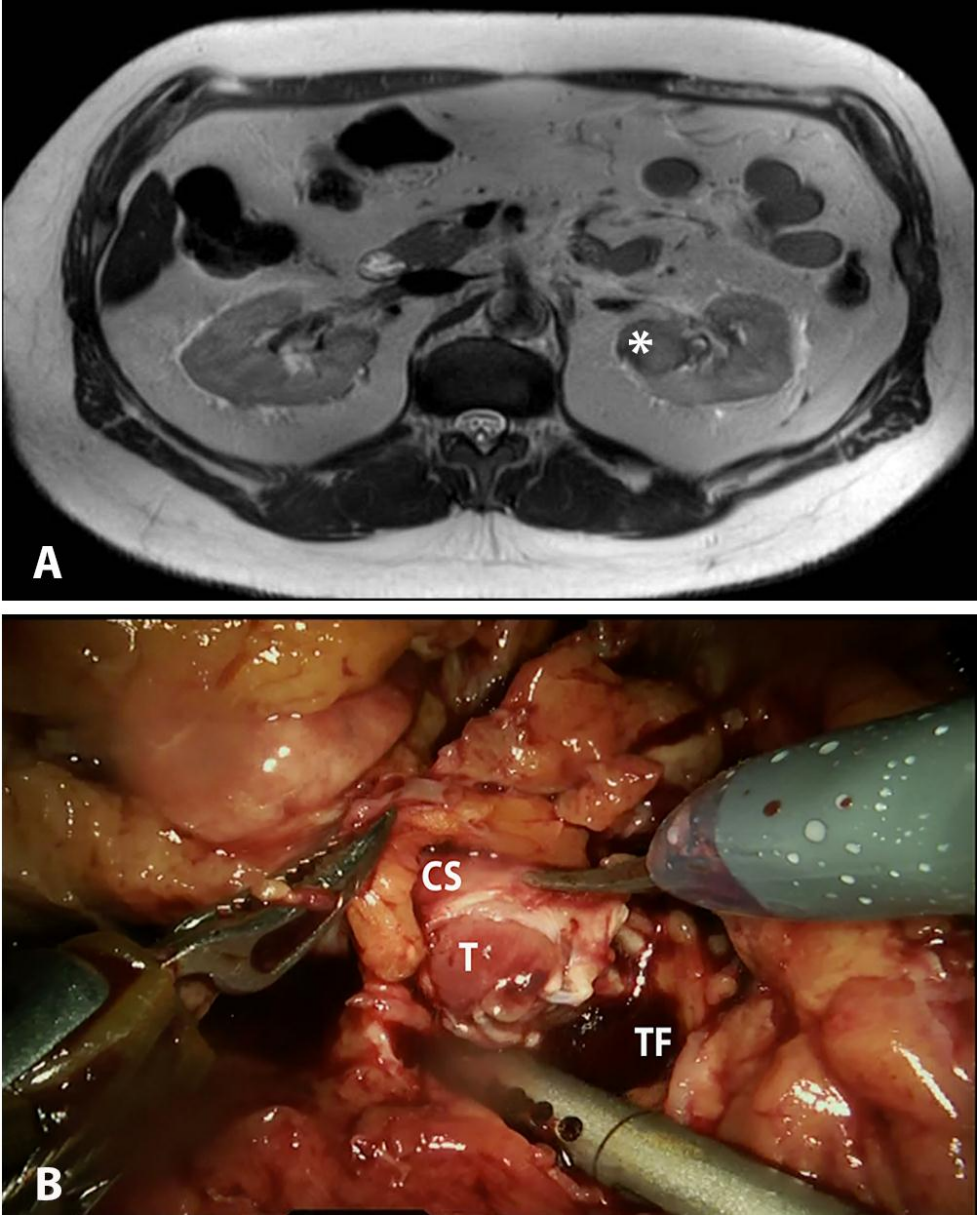
**A,** Magnetic resonance imaging (MRI) showing endophytic, posterior upper pole left renal mass (indicated by *), suspicious for carcinoma. **B,** Intraoperative photograph showing grossly invasive mass involving the lumen of the collecting system. T – Tumor; CS – collecting system; TF – tumor fossa.

We present a 62-year-old man with a history of hypertension and obesity who developed gross hematuria after a fall from his bicycle. When the hematuria persisted, magnetic resonance imaging (MRI) and computed tomography (CT) were performed, revealing a 3.6 x 3.2 x 2.5 cm left upper pole renal mass **(Figure 1 A)**. The mass was mostly endophytic, though still present at the cortico-medullary junction, so RCC and urothelial carcinoma (UC) were both potential diagnoses.

Left retrograde pyelogram and ureteroscopy performed one month prior to definitive surgery were normal, and selective cytology and brush biopsy were both negative for malignant cells. Chest CT was negative for metastatic disease. Given this workup, robotic partial nephrectomy (RPN) was performed for presumed endophytic RCC.

The kidney was fully mobilized and the main renal artery was clamped, then extirpation was attempted. However, upon entering the collecting system, the tumor was found to be inside the lumen **(Figure 1 B)**. The immediate concern was for UC, and the procedure was converted to a nephroureterectomy. The patient recovered from surgery and was discharged from the hospital on post-operative day two.

**Figure 2.**
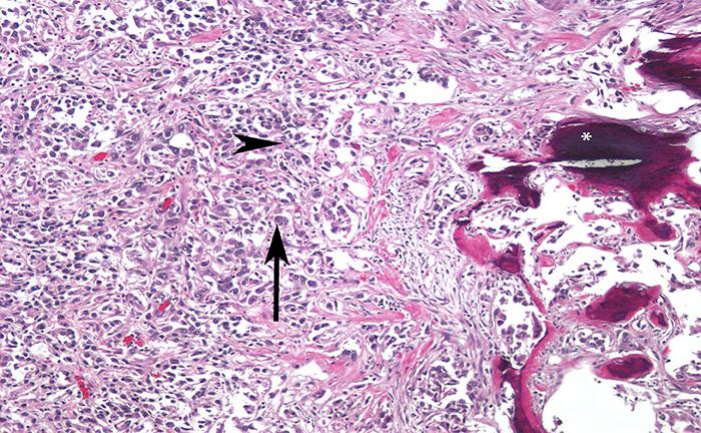
High magnification photomicrograph showing highly infiltrative carcinoma with tubular structure, embedded in desmoplastic stroma. The tumor cells have eosinophilic cytoplasm (arrowhead), the nuclei are large and pleomorphic, with prominent nucleoli (arrow) and coarse chromatin. Also seen is evidence of ossification (*).

On gross pathologic analysis, a yellow-tan, circumscribed and lobulated mass measuring 4.2 x 3.5 x 2.7 cm was found in the cortico-medullary junction of the upper pole. A pale tan tumor thrombus was identified in the renal pelvis, while no thrombus was identified in the renal vein. On microscopic examination, focal necrosis and multiple foci of osseous metaplasia were noted **(Figure 2)**. On immunohistochemistry, tumor cells were positive for PAX8, focally positive for CA-IX, and largely negative for CK903, p63, and GATA3. These findings are consistent with collecting duct carcinoma with sarcomatoid differentiation. The tumor was found to be invading the renal pelvis, renal cortex and perinephric fat; the sinus fat and renal vasculature were uninvolved. Thirty-five lymph nodes were removed, and seven were found to contain metastatic cancer.

As the patient presently has no signs of metastatic disease, he will be followed closely with imaging. Should he suffer from recurrence, he will likely undergo chemotherapy or combination immunotherapy with targeted therapy.

## Discussion

We present a rare case of a CDC that progressed from noninvasive to collecting system invasion within one month. CDC is a form of RCC known for its aggressive nature, and is still poorly understood due to its rarity. It is a malignant epithelial tumor that is derived from the principal cells of the collecting duct of Bellini, which is part of the renal collecting system. This is in contrast to the majority of RCCs, which are derived from the cells of the proximal convoluted tubules of the nephron. This is also distinct from urothelial carcinoma (UC), which arises from transitional epithelium of the bladder, renal pelvis and ureter. It accounts for <1% of all renal malignancies, and can affect ages 13-83-years old with a mean age of 55 years. It has a male to female ratio of 2:1 (8); 63.3% of patients are white, 27.5% are African American, and 9.2% are other races, according to a large retrospective study by Pepek et al. (9).

Potential symptoms at presentation include abdominal pain, hematuria, weight loss or flank mass; or patients may be asymptomatic (3, 4, 8). Case reports have described CDC associated with deep vein thrombosis, extensive coagulative necrosis, syndrome of inappropriate antidiuretic hormone secretion (SIADH) or leukocytosis secondary to increased granulocyte-colony stimulating factor (G-CSF) production; however these cases are atypical (5, 6). About one out of every three patients has metastases on presentation, and metastases to bone frequently are osteoblastic (8). Our patient presented with hematuria but had no signs of metastasis.

CDC is a pathologic diagnosis. The diagnosis is made if 1, at least some of the lesion involves the medullary region; 2, there is a predominant formation of tubules; 3, a desmoplastic stromal reaction is be present; 4, cytologic features are high grade; 5, growth pattern is infiltrative; and 6, there is an absence of other typical RCC subtypes or UC (8). Differentiation between CDC and UC can be challenging, but the addition of GATA3 to the immunohistochemical profile of p63 and PAX8 can help discriminate (10). Moreover, a recent study was able to detect distinct genetic differences between CDC and UC, concluding that CDC indeed has a distinct genetic pattern compared to UC. This study observed that CDC was associated with chromosomal DNA losses at 8p, 16p, 1p and 9p and gains in 13q, while UC was associated with loss at 9q, 13q, 8q and gains at 8p (11).

While a pathologic diagnosis is necessary for CDC, several studies have focused on imaging techniques that may help lead to early diagnosis. A study pooling 18 cases of proven CDC documented recurring CT findings. The mean longest diameter of the tumor was 6.9 cm and tumors were frequently solid, with a medullary location, weak or heterogeneous enhancement and infiltrative growth. Vascular invasion only occurred in 28% of cases (12). Unfortunately, these CT findings are nonspecific and cannot differentiate CDC from RCC.

In our case, UC was suspected given the location of the mass on imaging. However, since ureteroscopy and brush biopsy were both negative for abnormalities, the possibility of RCC involving the collecting system was not considered likely. It is speculated that the mass progressed to collecting system involvement rapidly after ureteroscopy, or that the preoperative imaging inadequately characterized the mass. There are no descriptions of this clinical scenario in the literature, though there is one case of CDC diagnosed by positive cytology (13).

The prognosis of CDC is poor as it is an aggressive disease, and one third of cases are metastatic on presentation (1). Pepek et al. found that three-year survival rates for localized, regional and distant disease were 93%, 45% and 6% respectively (9). Though several treatment options have been implemented with some success, a standard chemotherapy regimen is not yet established due to the rarity of CDC. Recently, Dason et al. conducted a systematic review of the management of CDC (14). The authors were able to identify three relevant studies, and concluded that a gemcitabine-cisplatin or gemcitabine-carboplatin regimen offered the best response.

Oudard et al. conducted a prospective multicenter phase II study evaluating gemcitabine-cisplatin/carboplatin in 23 patients with CDC. The authors reported a 26% partial/complete response rate (1 complete response), while another 10 patients (44%) experienced disease stabilization, and 7 (30%) had disease progression (15). Immunotherapy, in the form of interferon (IFN) and interleukin-2 (IL-2), has been studied and found to be ineffective (2, 16). In a large retrospective study of CDC patients from four Japanese institutions, there was no response 34 patients who received IFN or IL-2 (2).

Targeted therapy has also been evaluated in several very small trials. Procopio et al. (17) conducted a retrospective study of seven patients receiving targeted therapy, and identified two patients who lived for 49 months (sorafenib then sunitinib) and 19 months (temsiroliumus, then sunitinib). Though several other smaller studies exist (18–20), there is simply not enough data to provide a definitive answer on the efficacy of targeted therapy in CDC.

## Conclusion

We present a case of CDC that progressed to collecting system invasion in a short period of time and required radical nephrectomy. CDC is an aggressive form of RCC, associated with low 3-year survival and poor response to adjuvant therapy. While CDC is rare, it should be considered in those cases in which the clinical data seem incongruent with the imaging findings. Any surgeon attempting a partial nephrectomy should be aware of the possibility of surprise collecting system involvement, and should be prepared to revise the surgical plan as necessary.
